# Prevalence of fatigue functional and social impairment among patients with rheumatic diseases compared to patients without: A cross-sectional comparison

**DOI:** 10.1097/MD.0000000000033151

**Published:** 2023-03-03

**Authors:** Haya M. Almalag, Ibrahim Almaghlouth, Rufaidah Dabbagh, Abdulaziz R. Alsalem, Fahad N. Alrajban, Saleh A. Algarni, Faisal N. Alosaimi, Meshal I. Alassaf, Muhammad A. Alshamrani, Sulaiman Alzomia, Boshra Alanazi, Tareq Alalwan, Abdulaziz Alkhalaf, Mohamed Bedaiwi, Mohammed A. Omair

**Affiliations:** a Department of Clinical Pharmacy, College of Pharmacy, King Saud University, Riyadh, Saudi Arabia; b Rheumatology Unit, Department of medicine, College of Medicine, King Saud University, Riyadh, Saudi Arabia; c Department of Family and Community Medicine, College of Medicine, King Saud University, Riyadh, Saudi Arabia; d College of Medicine, King Saud University, Riyadh, Saudi Arabia.

**Keywords:** fatigue, functional, patient-reported outcomes, rheumatic diseases, Saudi Arabia, social impairment

## Abstract

Rheumatic diseases (RD) are chronic diseases that significantly affect the lives of patients. Assessing health outcomes through a patient-reported outcome measurement information system (PROMIS) is essential for RD management. Moreover, these tend to be less favorable among individuals than among the rest of the population. This study aimed to compare PROMIS between RD patients and other patients. This cross sectional study was conducted in the year 2021. Information about patients with RD was obtained from the RD registry at King Saud University Medical City. Patients without RD were recruited from family medicine clinics. Patients were contacted electronically through WhatsApp^©^ to complete the PROMIS surveys. We compared the individual PROMIS scores between the 2 groups using linear regression, adjusting for sex, nationality, marital status, education level, employment, family history of RD, income, and chronic comorbidities. There were 1024 individuals (512 with RD and 512 without RD). The most common RD was systemic lupus erythematosus (51.6%), followed by rheumatoid arthritis (44.3%). Individuals with RD reported significantly higher PROMIS T-scores for pain [β = 6.2; 95% confidence interval (CI) = 4.76, 7.71] and fatigue (β = 2.9; 95% CI = 1.37, 4.38) compared to those without RD. Moreover, RD individuals reported lower physical functioning (β = −5.4; 95% CI = −6.50, −4.24) and social interaction (β = −4.5; 95% CI = −5.73, −3.20). Patients with RD in Saudi Arabia, particularly those with systemic lupus erythematosus and rheumatoid arthritis, have significantly greater impairment in physical functioning and social interaction and report higher levels of fatigue and pain. Addressing and ameliorating these negative outcomes is necessary to improve quality of life.

**Key points**

- Patients with RD have significantly greater impairment in physical functioning and social interaction.
- Patients with RD report higher levels of fatigability and pain.
- Sex, nationality, marital status, level of education, employment, family history of RD, income, and chronic comorbidities were all confounding variables.

## 1. Introduction

Rheumatic diseases (RD) are lifelong chronic illnesses that have been subject to increased prevalence in recent years.^[1]^ The impact of these illnesses on patients extends far beyond the biomedical component, and affects their quality of life and wellness.^[2–4]^ Many patients with RD experience pain, disability, and social impairments.^[5]^ Biological, socioeconomic, and lifestyle choices can also contribute to the impairment in the quality of life of these individuals.^[6]^

There is a growing need to appropriately quantify the impact of RD on patient quality of life. Such assessments usually focus on several domains of patients’ lives, including psychological well-being, pain, functional disability, and social interaction, and use both generic and disease specific self-administrative tools.^[7]^ These tools vary in validity, ease of use, and comprehensiveness. Among these tools is the patient-reported outcomes measurement information system (PROMIS), which was developed to assess different aspects of patient-reported outcomes (PRO), such as pain, fatigue, physical functioning, and social interaction. PROMIS is used in patients with RD in many international settings, and is considered a good tool for enhancing the understanding of different PROs in the clinical management of RD.^[8]^

In Saudi Arabia, RD is a growing area of research interest. The prevalence of the most common RDs, rheumatoid arthritis (RA) and systemic lupus erythematosus (SLE), was explored and reported at 2.2 cases per 1000 population and 19 cases per 100,000 population, respectively.^[9,10]^ However, research on other RDs in Saudi Arabia is lacking. Recently, PROs research in patients with RD has increased in Saudi Arabia, with interesting findings compared to global reports.^[11–13]^ However, aspects of pain, fatigue, physical functioning. and social interactions in the RD have not been sufficiently explored in the Saudi population. Expanding the knowledge on PROs in relation to RD is of great importance to the medical and clinical rheumatological communities to improve the management of RD towards a more holistic and patient-centered approach. Therefore, this study aimed to measure PROs among individuals with RD at one of the largest tertiary medical centers in Saudi Arabia and compare them with the PROs of patients without RD.

## 2. Methods

### 2.1. Study design and settings

This cross sectional study was conducted in 2021 at King Saud University Medical City (KSUMC), in accordance with the guidelines for strengthening observational studies in epidemiology.^[14]^ KSUMC is a tertiary university hospital in Riyadh, whose beneficiaries are mostly the King Saud University faculty, students, and staff, along with their dependents, in addition to a few individuals referred from other governmental hospitals.

### 2.2. Study participants

As the purpose of our study was to compare PROs between individuals with RD and those without RD, we sampled equal proportions of patients with RD and those without RD from the KSUMC patient lists. Patients with RD were recruited from a clinical registry. This registry was first initiated in March 2020, and includes patients diagnosed with RD by a charitable association for RD, the Saudi Inflammatory Disease Patient Support Group, and specialized rheumatology clinics at KSUMC. Data collection for this registry is ongoing at the time of writing this manuscript. RD-free patients were recruited from a family medicine clinic. Patients mobile numbers were identified and randomly selected from the list. Patients were invited to participate in this study using text messages sent via WhatsApp^©^ (WhatsApp LLC, USA). Electronic informed consent was obtained and self-administered questionnaires were sent to the participants.

### 2.3. Sample size

We estimated the sample size with reference to a study conducted by Nagaraja et al^[8]^, which assessed standardized PROMIS scores for individuals with rheumatological conditions. In that study, the mean T-scores were 40 (±8) for physical functioning, 58 (±10) for fatigue, 47 (±9) for ability to participate in social roles, and 55 (±8) for pain behavior. Considering these assumptions and assuming a desired precision of 1, the respective sample sizes required to achieve a confidence level of 95% would be 246 for estimating physical functioning PROMIS scores, 385 for fatigue PROMIS scores, 312 for the ability to participate in social roles PROMIS scores, and 246 for pain intensity PROMIS scores. Therefore, a minimum sample size of 385 participants per group was required to calculate PROMIS scores for all 4 domains. Assuming a response rate of 75%, the required sample size for each group is 514.

### 2.4. Study variables and measurements

#### 2.4.1. Study outcomes.

In this study, the outcome measures were the PROMIS scores for fatigue, physical functioning, pain, and social interaction. These were measured using questions from the 29-item PROMIS profile version 2.1, which assessed these 4 domains.^[15]^ This tool consists of 8 items pertaining to fatigue, 10 items pertaining to physical functioning, 3 items pertaining to pain intensity, and 3 items pertaining to social interaction. The pain questions were scored on a 10-point Likert scale, while the other domains were scored on a 5-point Likert scale. For each domain, item scores were summed to create 4 separate composite scores. Finally, each of these composite scores was transformed into a T-score using the metric system website for PROMIS for each patient (https://www.assessmentcenter.net/ac_scoringservice). The score was calibrated using an average US score of 50 and a standard deviation of 10 as the reference group. The T-score was then categorized according to the PROMIS standardized cutoff points on the health measures website as normal, mild, moderate, and severe for fatigue, pain, and physical functioning, while social interaction was categorized as very high, high, average, low, and very low.

### 2.5. Study exposures

The survey also collected data on age, sex, marital status, nationality, education level, employment status, monthly household income in Saudi Riyals (SAR), family history of RD, and comorbidities.

### 2.6. Data collection

The data were collected electronically using Google Forms. Participation in the survey was voluntary. Patient identifiers were excluded from this study.

### 2.7. Ethical approval

The study was approved by the ethical approval of King Saud University Institutional Review Board No. E-20-4787), and information was managed with extreme confidentiality, without any participant’s identifiers in the data collection form. In addition, the study complied with the ethical standards of the Declaration of Helsinki and all participants provided written informed consent.

### 2.8. Statistical analysis

Data analyses were performed using IBM statistical package for social sciences (SPSS) Statistics version 27.0 (IBM, Armonk, NY).^[16]^ We calculated the means and standard deviations for age and PROMIS scores were calculated, whereas frequencies and percentages were calculated for categorical measures. Categorical variables were compared between the 2 groups (patients with RD vs patients without RD) using chi-square tests, whereas continuous measures were compared using independent *t* tests. Finally, the association between RD status and PROMIS was assessed using linear regression analyses, in which each PROMIS sub score and t score were added as dependent variables, while RD status and covariates were added as independent variables. For these models, we controlled for sex, nationality, marital status, education level, employment, family history of RD, income, and chronic comorbidities. An alpha level of 0.05 was used for significance testing. Data was done by complete case analysis.

## 3. Results

A total of 1024 participants were included in the study, with 512 in each group. The response rates were 85% for patients with RD and 92.4% for patients without RD. For comorbidities of participants with or without RD, are available in supplementary material Table S1, Supplemental Digital Content, http://links.lww.com/MD/I575. Participant without RD attended clinics for different disorders including diabetes mellitus (significantly higher than patients with RD), chronic obstructive pulmonary disease, peptic ulcer disease and peripheral vascular disease. Disorders that were significantly common in participants with RD were: fibromyalgia, chronic obstructive pulmonary disease, other connective tissue disease, medullary cystic kidney disease, and cerebrovascular accident. RDs were distributed as follows: 264 (51.6%) cases of systemic lupus erythematosus, 227 (44.3%) rheumatoid arthritis, 7 (1.4%) scleroderma, 3 (0.6%) Wegner granulomatosis, 2 (0.4%) Bechet’s disease, 2 (0.4%) polychondritis, 2 (0.4%) Sjogren’s syndrome, 1 (0.2%) spondylarthritis, 1 (0.2%) sarcoidosis, 1 (0.2%) gout, 1 (0.2%) antiphospholipid syndrome, and 1 (0.2%) case of vasculitis. Overall, the mean age was the same in both groups. However, most of the participants in the RD group were female. Moreover, individuals with RD differed significantly from the other patients in terms of sociodemographic characteristics (Table 1).

**Table 1:** Participants’ demographic characteristics.

		Rheumatologic disease (RD)				*P* value
		No N = 512		Yes N = 512		
		Count	Count	Count	Count	
Age in yr mean (Sd)		40 (14)		41 (13)		.350
Gender	Male	264	51.0%	66	12.0%	<.001*
	Female	248	48.0%	446	87.1%	
Nationality	Saudi	479	93.6%	496	96.9%	<.001*
	Other	33	6.4%	16	3.1%	
Marital status	Married	390	76.2%	303	59.2%	<.001*
	Single	97	18.9%	134	26.2%	
	Separated, widowed	25	4.9%	75	14.6%	
Level of education	Less than primary	5	1.0%	24	4.7%	<.001*
	Primary school	11	2.1%	29	5.7%	
	High school	149	29.1%	130	25.4%	
	Diploma	62	12.1%	61	11.9%	
	Bachelor’s	221	43.2%	217	42.4%	
	Masters or doctorate	64	12.5%	51	10.0%	
Employment	Student	58	11.3%	43	8.4%	<.001*
	Homemaker	81	15.%	169	33.%	
	Manual labor	25	4.9%	33	6.4%	
	Office worker	238	46.%	161	31.%	
	Retired	74	14.5%	37	7.2%	
	Unemployed	36	7.0%	69	13.5%	
Monthly household income (SAR)	< 5000	80	15.6%	136	26.6%	<.001*
	5000 to10,000	185	36.1%	165	32.2%	
	10,000 to < 15,000	114	22.3%	101	19.7%	
	15,000 to < 20,000	67	13.1%	54	10.5%	
	20,000 to < 30,000	34	6.6%	32	6.3%	
	>30,000	32	6.3%	24	4.7%	
Comorbid condition†	No	331	64.6%	254	49.6%	<.001*
	Yes	181	35.4%	258	50.4%	
Family history of RD	No	392	76.6%	335	65.4%	
	Yes	120	23.4%	177	34.6%	<.001*

RDSAR = Saudi Riyals, SD = slandered deviation.

* Significant according to a level of < 0.05.

† Comorbid conditions included diabetes, congestive heart failure, cerebrovascular disease, peripheral vascular disease, peptic ulcer disease, liver disease, chronic obstructive lung disease, dementia, cancer and hemiplegia.

**Figure 1.**
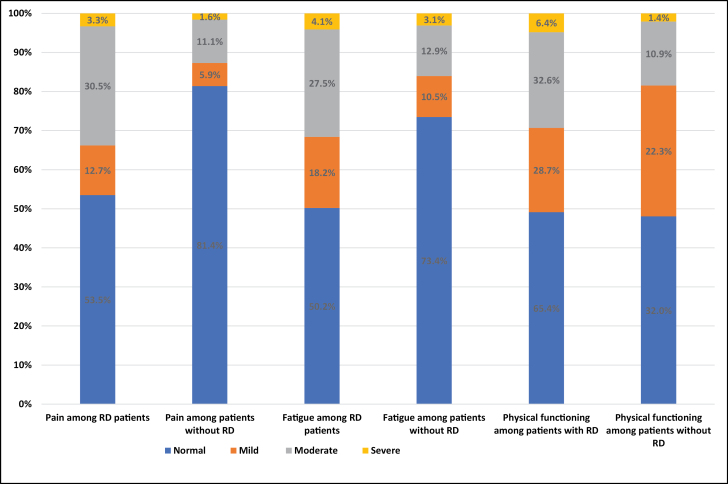
Distribution of pain, fatigue and physical functioning PROMIS levels among rheumatic diseases (RD) patients and those without RD. PROMIS = patient-reported outcome measurement information system.

**Figure 2.**
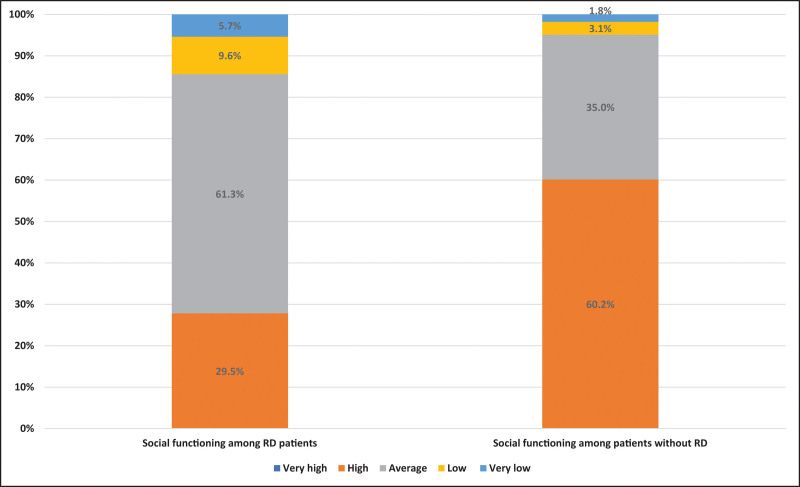
PROMIS levels of social interaction among patients with rheumatic diseases (RD) and those without RD. PROMIS = patient-reported outcome measurement information system.

Overall, the PROMIS sub-scores for pain and fatigue were significantly higher among patients with RD than among those without RD; the *P* value for independent *t* tests was < 0.001. Additionally, individuals without RD reported higher PROMIS sub-scores for physical functioning and social interaction (*P* < .001) (Table 2). The percentage of “severe” levels of pain, fatigue, and physical functioning were greater among patients with RD (Fig. 1). Furthermore, the percentage of very low social interaction was greater among these patients, reflecting a poorer quality of life among patients with RD than among those without RD (Fig. 2).

**Table 2:** Patient-reported outcomes among the participants.

	Rheumatologic disease (RD)				*P* value
	No N = 512		Yes N = 512		
	Mean	SD	Mean	SD	
PROMIS^©^ Pain	5	3	7	3	<.001*
PROMIS^©^ Pain t-score	43.8	10.61	52.6	11.08	<.001*
PROMIS^©^ Fatigue	17	9	21	9	<.001*
PROMIS© Fatigue t-score	47.6	11.57	53.6	10.88	<.001*
PROMIS^©^ Physical function	44	7	38	9	<.001*
PROMIS© Physical function t-score	49.6	7.99	42.4	8.68	<.001*
PROMIS^©^ Social	35	7	28	9	<.001*
PROMIS^©^ Social t-score	55.6	8.53	49.3	9.86	<.001*

PROMIS = patient-reported outcomes measurement information system, SD = stander deviation.

* *P* value for independent Student *t* test, significant at 0.05.

Multivariate linear regression analyses revealed that sub-scores and T-scores for pain and fatigue were positively associated with RD, while sub-scores and T-scores for physical activity and social interaction were negatively associated with RD. More precisely, controlling for other covariates, RD was associated with 6.2 points increase in the T-score for pain [95% confidence interval (CI) = 4.76, 7.71], 2.9 points increase in the T-score for fatigue (95% CI = 1.37, 4.39), 5.4 points decrease in the T-score for physical functioning (95% CI = −6.50, −4.24), and 4.5 points decrease in the T-score for social interaction (95% CI = −5.73, −3.20) compared to those without RD (Table 3).

**Table 3:** Multiple linear regression for the association between rheumatologic disease status (independent variable) and PROMIS scores (dependent variable).

	Unadjusted beta	95% confidence intervals	*P* value	Adjusted beta†	95% confidence intervals	*P* value
PROMIS© Pain	2.1	1.79 to 2.46	<.001*	1.5	1.11 to 1.86	<.001*
PROMIS© Pain T-score	8.8	7.45 to 10.11	<.001*	6.2	4.76 to 7.71	<.001*
PROMIS© Fatigue	4.6	3.56 to 5.67	<.001*	2.0	0.86 to 3.13	.001*
PROMIS© Fatigue T-score	6.3	4.88 to 7.63	<.001*	2.9	1.37 to 4.39	<.001*
PROMIS© Physical function	−6.5	−7.55 to −5.52	<.001*	−4.8	−5.89 to −3.63	<.001*
PROMIS© Physical function T-score	−7.2	−8.23 to −6.18	<.001*	−5.4	−6.50 to −4.24	<.001*
PROMIS© Social interactions	−6.3	−7.27 to −5.23	<.001*	−4.5	−5.60 to −3.33	<.001*
PROMIS© Social interactions T-sore	−6.3	−7.43 to −5.16	<.001*	−4.5	−5.73 to −3.20	<.001*

PROMIS = patient-reported outcomes measurement information system

* Significant according to level of<0.05

† Adjusted beta for sex, nationality, marital status, region, level of education, employment, family history, income, and chronic comorbidities.

## 4. Discussion

The burden of RD is reflected in PROs, which include fatigue, physical function, and social interaction. This finding was also observed in the present study. The findings of our study can be summarized as follows: First, PROMIS scores were significantly poorer among patients with RD than among those without RD. Second, even when controlling for potential confounding factors, RD was positively associated with higher PROMIS sub scores for pain and fatigue and negatively associated with higher PROMIS sub scores for physical functioning and social interaction, all of which suggest a significantly poorer quality of life among individuals with RD compared to the rest of the population.

A recent large time series event study during the COVID-19 pandemic highlighted the prevalence of impairment in psychosocial and functional domains among patients with inflammatory arthritis living in Saudi Arabia.^[12,13]^ However, it is unclear whether this degree of impairment differs significantly from that in the general population. Therefore, this significant difference was reflected in the present study.

SLE and RA comprise most patients with RD, which is consistent with the 2 most common inflammatory arthritis conditions in Saudi Arabia and globally.^[12]^ Most of the patients were female, with significant differences in employment, income, and marital status, favoring the general population. This is particularly important, given the impact of social determinants on health and patient-reported outcomes. In support of this notion, a recent study by Izadi et al^[17]^ showed that social disparity is associated with higher functional decline in patients with RA, which is partially mediated by disease activity.

As expected, the pain score was much higher in the patients with RD. Pain is a complex phenomenon, and the sources of pain can be multifactorial.^[18]^ Obviously, Disease activity is a key factor. However, other centrally driven causes, such as fibromyalgia, which was more commonly observed in our cases, tended to be another important factor.^[19]^ Pain also tends to be more prevalent in patients with co morbidities. Our data supports these findings. Identifying all potential factors associated with pain among patients with RD is paramount to understanding their pain, which significantly impairs their quality of life and likely affects other aspects of their quality of life, such as physical function, social interaction, and even mood.^[13]^ In addition, one should keep in mind other contributing factor that may affect physical function and social interaction as the chronicity of rheumatic diseases that is Disease duration.^[20,21]^

Fatigue is more prevalent in patients with RD than in the general population. In this study, Saudi patients with RD experienced significantly more fatigue, affecting almost 50% of the patients in the general population. Furthermore, almost 1-third of patients living with RDs experience moderate to severe fatigue, which is likely to affect their engagement with society and productivity at work.^[22]^ This was evident in the close relationship between fatigue and social interaction scores, as well as the higher prevalence of impairment in social interaction among rheumatic patients compared to the general population. In a qualitative assessment of the impact of fatigue on patients with SLE by Sterling et al^[23]^, work and family engagement were frequently found to be affected in patients with SLE.

With a high burden of fatigue, impaired social interaction, and increased prevalence of pain and fibromyalgia, it is not surprising to see significant impairment in physical function. In our recent work on the impact of the COVID19 pandemic on the quality of life of patients with RD in Saudi Arabia, a very tight relationship between fatigue, physical function, and social interaction exists, such that 1 may influence the other. Physical function has also been associated with the development and worsening of multimorbidity, which is more prevalent among patients with RD. Hence, it is crucial to address exercises even in their simple and regular forms in patients suffering from RD, knowing that the benefits extend to various domains including mood, metabolism, pain, and even chronic inflammation.

This study was conducted to address the unmet need to understand the burden of RD, particularly RA and SLE, in patients compared with the general population. Both cases and controls were sampled from the same pool of populations, and the tools used to assess patient-reported outcomes were validated and universally used. However, this study has some limitations, including unmeasured confounders, such as disease activity or formal assessment of mood disorders. This must be considered during the interpretation of regression models. In conclusion, this study sheds light on the burden of RDs in patients compared with that in the general population. Patients with RDs have impaired physical function, social interactions, fatigue, and pain. These findings are consistent with global reports and highlight the need for population-based interventions to improve the quality of life.

## Acknowledgements

We would like to thank the patients and Charitable Association for Rheumatic Diseases for their assistance and support.

## Author contributions

**Conceptualization:** Ibrahim Almaghlouth, Rufaidah Dabbagh, Abdulaziz Alkhalaf, Mohamed Bedaiwi, Mohammed A. Omair.

**Data curation:** Haya M. Almalag, Ibrahim Almaghlouth, Rufaidah Dabbagh, Abdulaziz R. Alsalem, Fahad N. Alrajban, Saleh A. Algarni, Faisal N. Alosaimi, Meshal I. Alassaf, Muhammad A. Alshamrani, Sulaiman Alzomia, Boshra Alanazi, Tareq Alalwan, Abdulaziz Alkhalaf, Mohamed Bedaiwi, Mohammed A. Omair.

**Formal analysis:** Haya M. Almalag, Ibrahim Almaghlouth, Rufaidah Dabbagh.

**Investigation:** Abdulaziz R. Alsalem, Fahad N. Alrajban, Saleh A. Algarni, Faisal N. Alosaimi, Meshal I. Alassaf, Sulaiman Alzomia, Boshra Alanazi, Tareq Alalwan.

**Methodology:** Haya M. Almalag, Ibrahim Almaghlouth, Muhammad A. Alshamrani.

**Software:** Haya M. Almalag.

**Supervision:** Abdulaziz Alkhalaf, Mohamed Bedaiwi, Mohammed A. Omair.

**Visualization:** Haya M. Almalag.

**Writing – original draft:** Haya M. Almalag, Ibrahim Almaghlouth.

**Writing – review & editing:** Haya M. Almalag, Ibrahim Almaghlouth, Rufaidah Dabbagh, Abdulaziz R. Alsalem, Fahad N. Alrajban, Saleh A. Algarni, Faisal N. Alosaimi, Meshal I. Alassaf, Muhammad A. Alshamrani, Sulaiman Alzomia, Boshra Alanazi, Tareq Alalwan, Abdulaziz Alkhalaf, Mohamed Bedaiwi, Mohammed A. Omair.

## Supplementary Material



## References

[1] Lerner A Jeremias P Matthias T. The world incidence and prevalence of autoimmune diseases is increasing. Int J Celiac Dis. 2016;3:151–5.

[2] Choi ST Kang JI Park I-H. Subscale analysis of quality of life in patients with systemic lupus erythematosus: association with depression, fatigue, disease activity and damage. Clin Exp Rheumatol. 2012;30:665–72. 22704691

[3] Pereira MG Duarte S Ferraz A. Quality of life in patients with systemic lupus erythematosus: the mediator role of psychological morbidity and disease activity. Psychol Health Med. 2020;25:1247–57. 32093519 10.1080/13548506.2020.1728350

[4] Macejová Z Záriková M Oetterová M. Systemic Lupus Erythematosus - Disease impact on patients. Cent Eur J Public Health. 2013;21:171–3. 24344545 10.21101/cejph.a3818

[5] Russell AS Gulliver WP Iriven EJ. Quality of life in patients with immune-mediated inflammatory diseases. J Rheumatol Suppl. 2011;88:7–19. 22045973 10.3899/jrheum.110899

[6] Figueiredo-Braga M Cornaby C Cortez A. Depression and anxiety in systemic lupus erythematosus. Medicine (Baltim). 2018;97:e11376. 10.1097/MD.0000000000011376 PMC6076116 29995777

[7] Gossec L. Patient-reported outcomes in rheumatoid arthritis: why are they important and how should they be assessed? Turkish J Rheumatol. 2010;25:99–104.

[8] Nagaraja V Mara C Khanna PP. Establishing clinical severity for PROMIS® measures in adult patients with rheumatic diseases. Qual Life Res. 2018;27:755–64. 28983738 10.1007/s11136-017-1709-z PMC5845827

[9] Al-Dalaan A Al Ballaa S Bahabri S. The prevalence of rheumatoid arthritis in the Qassim Region of Saudi Arabia. Ann Saudi Med. 1998;18:396–7. 17344708 10.5144/0256-4947.1998.396

[10] Al-Arfaj AS Al-Balla SR Al-Dalaan AN. Prevalence of systemic lupus erythematosus in central Saudi Arabia. Saudi Med J. 2002;23:87–9. 11938371

[11] Omair MA Alshehri MM Altokhais NA. Exploring factors influencing medication compliance in saudi rheumatoid arthritis patients: a nationwide cross-sectional survey - results from the COPARA study. Patient Prefer Adherence. 2022;16:1105–14. 35502428 10.2147/PPA.S363477 PMC9056069

[12] Hassen LM Almaghlouth IA Hassen IM. Impact of COVID-19 outbreak on rheumatic patients’ perceptions and behaviors: a cross-sectional study. Int J Rheum Dis. 2020;23:1541–9. 32940963 10.1111/1756-185X.13959

[13] Hassen LM Albarrak RA Albahlal RA. Functional and psychosocial impact of COVID-19 pandemic on rheumatic patients’ quality of life in Saudi Arabia. Qual Life Res. 2022;31:3229–39. 35857205 10.1007/s11136-022-03184-1 PMC9297668

[14] von Elm E Altman DG Egger M. The strengthening the reporting of observational studies in epidemiology (STROBE) statement: guidelines for reporting observational studies. J Clin Epidemiol. 2008;61:344–9. 18313558 10.1016/j.jclinepi.2007.11.008

[15] Cella D Riley W Stone A. The patient-reported outcomes measurement information system (PROMIS) developed and tested its first wave of adult self-reported health outcome item banks: 2005-2008. J Clin Epidemiol. 2010;63:1179–94. 20685078 10.1016/j.jclinepi.2010.04.011 PMC2965562

[16] IBM Corp. Released 2021. IBM SPSS Statistics for Windows 28.0. Armonk, NY: IBM Corp; 2021.

[17] Izadi Z Li J Evans M. Socioeconomic disparities in functional status in a national sample of patients with rheumatoid arthritis. JAMA Netw Open. 2021;4:e2119400. 34347058 10.1001/jamanetworkopen.2021.19400 PMC8339935

[18] Phillips K Clauw DJ. Review: central pain mechanisms in the rheumatic diseases: future directions. Arthritis Rheum. 2013;65:291–302. 23045168 10.1002/art.37739 PMC3610409

[19] Williams DA Clauw DJ. Understanding fibromyalgia: lessons from the broader pain research community. J Pain. 2009;10:777–91. 19638325 10.1016/j.jpain.2009.06.001 PMC2741022

[20] Santo RC do E Baker JF Dos Santos LP. Changes in physical function over time in rheumatoid arthritis patients: a cohort study. PLoS One. 2023;18:e0280846. 36689423 10.1371/journal.pone.0280846 PMC9870154

[21] Rodríguez-García E Barnes-Ortiz S Pérez-Mármol JM. Self-efficacy, pain intensity, rheumatic disease duration, and hand functional disability on activities of daily living. Nurs Res. 2020;69:E208–16. 32804710 10.1097/NNR.0000000000000466

[22] Kawka L Schlencker A Mertz P. Fatigue in systemic lupus erythematosus: an update on its impact, determinants and therapeutic management. J Clin Med. 2021;10:3996. 34501444 10.3390/jcm10173996 PMC8432566

[23] Sterling K Gallop K Swinburn P. Patient-reported fatigue and its impact on patients with systemic lupus erythematosus. Lupus. 2014;23:124–32. 24197552 10.1177/0961203313511554

