# Supplementary material for: Prevalence of fatigue functional and social impairment among patients with rheumatic diseases compared to patients without: A cross-sectional comparison
Source: Medicine (Baltimore). 2023 Mar 3;102(9):e33151. doi: 10.1097/MD.0000000000033151 (PMC9981388; doi:10.1097/MD.0000000000033151)
Supplement: Supplementary file 1 [file medi-102-e33151-s001.pdf]

Supplementary material table 1. Different comorbidities of included participants with and without rheumatic diseases

	**Without RD**	**%**	**With RD**	**%**	**Total**	**%**	***p* value**
Uncomplicated diabetes mellitus (DM)	**110**	**62.5**	66	37.5	176	17.2	<0.001*
Chronic obstructive pulmonary disease (COPD)	37	31.4	**81**	**68.6**	118	11.5	<0.001*
Peptic Ulcer	52	47.3	58	52.7	110	10.7	0.545
Other connective tissue disease (CTD)	0	0.0	**85**	**100.0**	85	8.3	<0.001*
Fibromyalgia	3	5.1	**56**	**94.9**	59	5.8	<0.001*
Medullary cystic kidney disease (MCKD)	4	7.4	**50**	**92.6**	54	5.3	<0.001*
Peripheral vascular disease (PVD)	11	36.7	19	63.3	30	2.9	0.138
Moderate Liver Disease	5	20.0	**20**	**80.0**	25	2.4	0.002*
Myocardial infarction (MI)	8	42.1	11	57.9	19	1.9	0.487
Cerebrovascular accident or transient ischemic attach (CVA/TIA)	3	16.7	**15**	**83.3**	18	1.8	0.004*
Local Solid tumor	8	57.1	6	42.9	14	1.4	0.789
Lymphoma	3	37.5	5	62.5	8	0.8	0.725
DM with end organ failure	2	33.3	4	66.7	6	0.6	0.687
Dementia	2	40.0	3	60.0	5	0.5	1.000
Severe Liver Disease	3	60.0	2	40.0	5	0.5	1.000
Hemiplegia	2	50.0	2	50.0	4	0.4	1.000
Congestive heart failure (CHF)	2	66.7	1	33.3	3	0.3	1.000
Leukemia	1	100.0	0	0.0	1	0.1	1.000

*Significant according to a level of <0.05; **Bold:** significantly higher percent
